# Anticancer Activity of *Rhizophora mucronata* Leaves Extract on Sprague–Dawley Rats: *In Vivo* Model

**DOI:** 10.1155/2023/6665012

**Published:** 2023-12-16

**Authors:** Asbin Mary X, Syed Ali Mohamed Yacoob, Anuradha Venkatraman, Ruban Packiasamy, Meivelu Moovendhan, Murugesan Gnanadesigan, Yogananth Nagarajan

**Affiliations:** ^1^PG Department of Biotechnology, Alpha College of Arts and Science, Porur, Chennai, Tamil Nadu, India; ^2^PG and Research Department of Biotechnology, Mohamed Sathak Arts and Science College, Chennai, Tamil Nadu, India; ^3^PG and Research Department of Biochemistry, Mohamed Sathak Arts and Science College, Chennai, Tamil Nadu, India; ^4^Department of Biotechnology, SNMV College of Arts and Science, Coimbatore, Tamil Nadu, India; ^5^Centre for Ocean Research, Sathyabama Institute of Science and Technology, Chennai, Tamil Nadu, India; ^6^Natural Products Research Laboratory, Department of Microbial Biotechnology, Bharathiar University, Coimbatore 641 046, Tamil Nadu, India; ^7^PG and Research Department of Microbiology, Mohamed Sathak Arts and Science College, Chennai, Tamil Nadu, India

## Abstract

Medicinal plants are now used to treat cancer due to the presence of bioactive compounds. Apart from the plants, mangroves also possess rich bioactive compounds with high medicinal activity. Based on the ethnobotanical attributes of *Rhizophora mucronata*, we are keen to work with its anticancer activity. The aim of the study is to assess the anticancer activity of methanolic extract of *Rhizophora mucronata* leaves against breast cancer. Its safety profile for anticancer investigations was therefore confirmed through an acute toxicity assessment. In accordance with OECD guiding principles, the study was approved to evaluate the toxicity, including acute and subacute effects and anticancer activities of methanolic extract of *Rhizophora mucronata* leaves on Sprague–Dawley rats. In acute toxicity trials, the dose of 1000 mg/kg body weight was determined to be safe and nontoxic even at high dose levels and did not result in any indicators of toxicity or death in the tested groups compared to controls for 14 days. In contrast, rats in a subacute toxicity study were given consistent doses of 100 mg/kg, 200 mg/kg, and 300 mg/kg for a total of 28 days along with a control group. Haematological, biochemical, and histological tests conducted in advance revealed that methanolic extract of *Rhizophora mucronata* leaves (MERML) at repeated doses of 200 mg/kg and 300 mg/kg was normal and had no significant effects on the treated groups. *Rhizophora mucronata* extract (250 mg/kg) was successfully used in *in vivo* trials to stop the growth of breast cancer cells in groups that had been given DMBA. Based on these findings, it has been concluded that methanolic extract of *Rhizophora mucronata* leaves (MERML) was safe at both higher and lower dosages and could be assessed for pharmacological study.

## 1. Introduction

The disease of tissue growth directive failure cancer is a vast collection of illnesses. The genes that control cell growth and division have to be distorted for a common cell to change into a tumour cell [1]. Cancer is the subject of intense anxiety and is forbidden, and 200 different varieties of cancer concern humans, despite the fact that many diseases may have a prognosis that is much worse than most cases of cancer [2]. Cancer has a wide variety of complex, poorly understood causes. Numerous factors, such as smoking, eating particular foods, contracting certain illnesses, being exposed to radiation, not engaging in sexual activity, being overweight, and environmental contaminants, are known to increase the risk of developing cancer [3].

Natural commodities from plants have been used for the treatment of diverse diseases from ancient times. *Rhizophora mucronata* belongs to the family *Rhizophoraceae*. It is commonly known as the loop root mangrove, red mangrove, and Asiatic mangrove. The natural habitats of *Rhizophora mucronata* are estuaries, tidal creeks, and flat coastal areas. The specific medicinal properties of *Rhizophora mucronata* depend on the part employed and finds its use to treat human health problems. Based on literature studies, *Rhizophora mucronata* was traditionally used to treat a variety of conditions, including leprosy, diabetes, hypertension, constipation, menstrual disorders, fever, haematuria, ulcers, haemorrhage, diarrhoea, nausea, and fever. The purpose of the present study plan was to examine the anticancer effectiveness of methanolic extract of *Rhizophora mucronata* leaves (MERML) in Sprague–Dawley rats that had mammary carcinoma caused by DMBA.

## 2. Materials and Methods

### 2.1. Sample Collection

*Rhizophora mucronata* leaves were collected, authenticated and dried in shade, and powdered and soaked in methanol for 7 days. 500 gms of powdered leaves of *Rhizophora mucronata* were extracted with 1500 ml of methanol solvents using the Soxhlet extraction method for 25 hrs. Then, the methanolic extract of *Rhizophora mucronata* leaves (MERML) was filtered to obtain resolute volume and analysed for further studies such as various phytochemicals and detection of bioactive compounds using the GC-MS analysis.

### 2.2. Experiment Animals

Healthy young female adult Sprague–Dawley rats used for acute, subacute, and toxicity studies, weighing 150–200 gm, aged between 6 and 8 weeks old weeks, were obtained from Biogen, Bangalore. The experiments were conducted at the Animal House Facility of C.L. Baid Metha College of Pharmacy, Department of Pharmacology, Thoraipakkam, Chennai-600 097, Tamil Nadu, India. The experimental research proposals were authorized by the Institutional Animal Ethics Committee (IAEC), CPCSEA, New Delhi, and received an approval IAEC no. 10/321/PO/Re/S/01/CPCSEA dated 11/10/2019. Also, the work was carried out through recent guidelines for the concern of laboratory animals. The rats were adaptive toward laboratory ambience for 7 days prior to carry out the research. The obtained rats were maintained in room temperature at 22°C ± 3°C under natural light/dark cycle, with relative moisture of slightest 30% and not more than 70%. During this process, rats were housed in cages and provided with pellet diet and clean water.

### 2.3. Acute Oral Toxicity Studies

The study [4] was performed using 12 female Sprague–Dawley rats divided into two groups and put into the cages. Group I (6 females) of SD rats were administrated with distilled water since they act as control group (Table 1). Also, to the Group II (6 females) of SD rats, the MERML extract is suspended in tween 80 and administered in an oral dose by gavage with a feeding pointer. The quantity stage of 1000 mg/kg body mass was managed as well as the nourishment for the rats is limited for more 3-4 hours. Besides the amount, the animals were checked for the parameters such as skin modification, mobility, assertive behaviour, and understanding sound and pain, as well as respiratory actions. Throughout the complete 14 days of study, the animals were observed closely for behaviour signs, body mass, and food and water intake.

### 2.4. Subacute Oral Toxicity Studies

Based on OECD guidelines-407, 28-day oral toxicity study was carried out in SD rats. In brief, 24 young Sprague–Dawley rats were selected with body weight of 150−200 gm and divided into four groups (Table 2). Six female animals are used for each group. Group I acts as the vehicle control and groups II, III, and IV are for the assessment of the MERML. The acute oral toxicity served as the basis for choosing the three dosing levels. The low dose (100 mg/animal), medium dose (200 mg/animal), and high dose (300 mg/animal) were calculated from the acute toxicity dose (1000 mg).

The rats were fed with standard food and water during the experiment. The studied animals were monitored daily for any changes in clinical indications or death after extract administration until the experiment was over. Every experimental rat's body weight was noted on days 1, 7, 14, and 28 of the study. Food and water consumptions were calculated for both the control and treatment groups in the same manner. Throughout the entire study, the mortality of all the animals in the control and test groups (low, mid, and high) was monitored twice a day. The animals were given isoflurane anaesthesia on the 28th day, and a necropsy study was carried out on every animal. For the biochemical investigations, blood samples were taken from the rat's orbital sinus region using sodium heparin (200 IU/ml). Additionally, blood samples were obtained for haematological examinations in potassium EDTA (1.5 mg/ml). Blood samples were centrifuged at 3000 rpm for 10 minutes and used for haematological and biochemical investigations. The organs such as the spleen, liver, and kidney were excised immediately and washed with ice-cold physiological saline and blotted dry. A part of the tissues from the spleen, liver, and kidney was removed and fixed in 10% formalin for histopathological study.

### 2.5. *In Vivo* Anticancer Studies

Four sets of Sprague–Dawley rats, each with six female animals, were used to study the anticancer effects of MERML extract on mammary carcinoma-induced animals. Group I animals serve as the vehicle control and Group II animals as mammary cancer inducers with DMBA 20 mg/kg body weight. Group III animals serve as the test administrated with MERML extract 250 mg/kg body weight. Each rat from Groups II and III receives a single dose of 7,12-dimethylbenz(a)anthracene (25 mg/kg body weight), which is mixed with 0.5 ml of olive oil with 0.5 ml of saline and administered through gastric intubation to cause breast cancer. The rats were also given a single dosage of alpha-tocopherol (200 mg/rat) following the intragastric administration of DMBA for 25 consecutive days [5]. Group IV animals act as positive control administrated with fluorouracil 20 mg/kg body weight (Table 3).

Each rat's body weight was recorded before being sacrificed at the end of the experiment. The heart puncture method was used to obtain the blood sample, which was then placed in centrifuge tubes with EDTA. After allowing the blood to coagulate, the serum was separated for biochemical and haematological analysis by centrifugation at 1000 g for 10 min at 4°C. The liver, kidney, and mammary tissue were immediately removed from sacrificed animals and carefully cleansed in ice-cold saline before being carefully blotted dry. For histological analysis, a tiny portion of the tissues from the liver, kidney, and mammary tissue was taken and preserved in 10% formalin.

#### 2.5.1. Haematological Investigations

Using a SYSMEX Kx-21 (Eraba, Transasia) automatic haematology analyzer, the whole blood sample was analysed for the estimate of haematological parameters such as haemoglobin (Hb), red blood cells (RBCs), white blood cells (WBCs), packed cell volume (PCV), and platelets count.

#### 2.5.2. Biochemical Investigations

The anticoagulated blood serums were analysed for biochemical parameters such as blood metabolite estimates for urea, creatinine, uric acid, protein, and albumin, as well as lipid profiling for total cholesterol and triglycerides. Additionally, measurements of enzymatic and nonenzymatic antioxidants, including the activities of catalase, glutathione peroxidase, superoxide dismutase, and lipid peroxidation (LPO), as well as liver function markers, such as aspartate transaminase and alanine transaminase, were studied.

#### 2.5.3. Histopathological Investigations

Haematoxylin and eosin staining was used for the histopathological research [6]. Histopathological tests were conducted on the dissected organs, such as the breast tissue, liver, and kidney, from all four groups of rats (Groups I, II, III, and IV). For 48 hours, the tissues were kept in 10% normal saline. Following a 24-hour period, the tissues were dehydrated by using ethyl alcohol-water with varying concentrations (50, 80, and 95%) before being incubated in alcohol. Also, xylene is used to cleanse the tissues from the alcohol. Also, next, using molten paraffin, the tissues were imbedded in the “*L*” moulds. The tissues were divided into sections of approximately 4–6 micron thickness using a rotary microtome. After cutting them into smaller pieces, the sections were stained with haematoxylin and eosin and mounted in neutral deparaffinization xylene (DPX) media for microscopic examination of the cells for any signs of necrosis and lipid alterations, etc. Axiostar plus microscope was used to take photographs with a Canon 10.1 megapixel digital camera from Japan (Zeiss, Germany).

### 2.6. Statistical Analysis

GraphPad Prism version 5 was used for all statistical calculations. The outcomes of the experiment were presented as mean SD. One-way analysis of variance (ANOVA) was used to examine the data, and then the post hoc Dunnett's test was employed to determine statistical significance.

## 3. Results

From Pichavaram Mangrove forest, fresh *Rhizophora mucronata* leaves were collected and authenticated with reference number PARC/2020/4352 by Prof. P. Jayaraman at Plant Anatomy Research Centre, West Tambaram, Chennai, Tamil Nadu. The *Rhizophora mucronata* leaves were cleaned, shade dried, powdered, and extracted using a methanol solvent by the Soxhlet extraction method. The yield of resultant extract (MERML) was 2.64 gm and used for further analysis. The extract has various phytoconstituents and the compound identification was recognized by using the GC-MS analysis, exposing nine compounds [7]. The stored extract was used for toxicity studies.

### 3.1. Acute Oral Toxicity Study

Group I rats served as the control group and Group II rats as the MERML treated group in the investigation on acute oral toxicity. The animals in Group II get dosages of MERML at high concentrations of 1000 mg/kg, and the animals in Group I serve as controls without extract administration. The behaviour of the Group I and Group II animals was tracked for 14 days. The animals in Groups I and II are closely monitored for any behavioural changes throughout the first four hours in both the test and control groups. The test groups given a high dose of 1000 mg/kg showed no toxicity, mortality, or behavioural abnormalities when compared to the control animals (Table 4).

**Figure 1:**
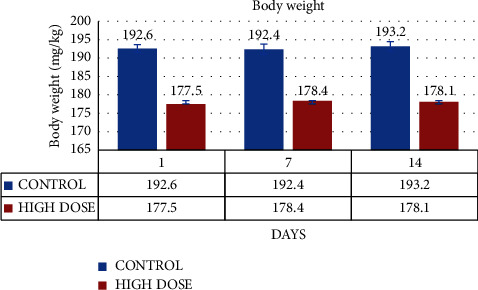
Graph shows the change in body weight (mg/kg) of SD rats exposed to methanol extract of *R. mucronata* leaves.

Animal body weights are recorded on days 1, 7, and 14 of the study. The experimental animal weight in the MERML test groups was studied on days 1, 7, and 14 at 177.5 ± 1.05 gm, 178.4 ± 1.2 gm, and 178.1 ± 3.1 gm, respectively. 192.6 ± 1.04 gm, 192.4 ± 1.4 gm, and 193.2 ± 1.3 gm were discovered in the control groups. The estimated and recorded weight changes are shown in Figure 1. Dunnett's test is employed as one type of ANOVA in the statistical analysis. However, throughout the course of the 14 days, there was no discernible difference (*p* < 0.05).

**Figure 2:**
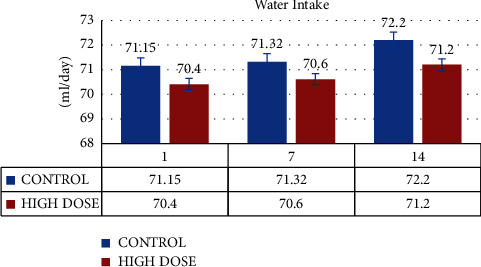
Graph shows the water intake (ml/day) of SD rats exposed to methanol extract of *R. mucronata* leaves (high dose-1000 mg/kg).

**Figure 3:**
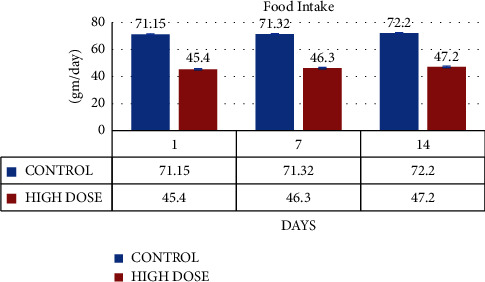
Graph shows the food intake (ml/day) of SD rats exposed to methanol extract of *R. mucronata* leaves (high dose-1000 mg/kg).

The water and food intake for the control and MERML test groups (Group I and Group II) was computed on days 1, 7, and 14, respectively. The Group II consumed 70.4 ± 2.3 ml of water on day 1, 70.6 ± 1.1 ml of water on day 7, and 71.2 ± 2.1 ml of water on day 14. Likewise, the control groups' water consumption was 71.5 ± 1.14 on day 1, 71.32 ± 1.13 on day 7, and 72.2 ± 1.1 on day 14. (Figures 2 and 3).

These results imply that a higher oral dose of 1000 mg/kg does not significantly increase the mortality of rats. The food and water intake is normal in both the test and control animals. This suggested that the MERML extract does not show toxicity in the animal groups.

### 3.2. Subacute Oral Toxicity Study

**Figure 4:**
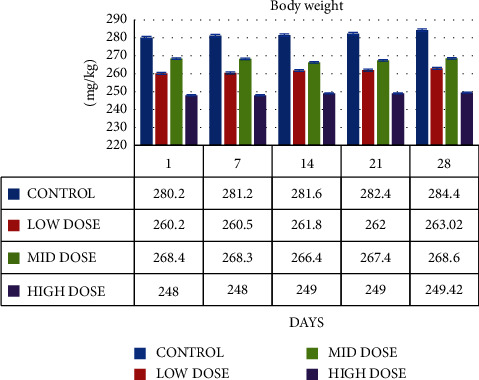
Graph shows the change in body weight (mg/kg) of SD rats exposed to methanol extract of *R. mucronata* leaves. Low dose, 100 mg/kg, mid dose, 200 mg/kg, and high dose, 300 mg/kg.

From day 1 through day 28 of subacute study, the rats' body weight decreased at concentrations of 100 mg/kg, 200 mg/kg, and 300 mg/kg in comparison to the control rats. The outcomes are displayed in Figure 4. The statistical analysis started with a one-way ANOVA method and finished with a Dunnett's test. However, throughout the course of the 28 days, there was no discernible difference (*p* > 0.05).

**Figure 5:**
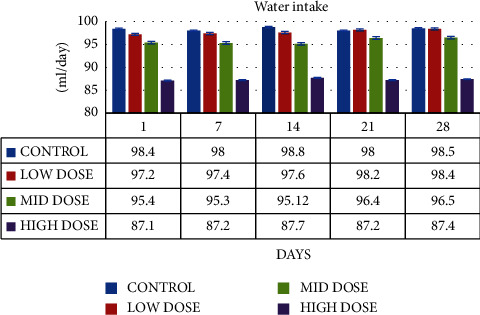
Graph shows the water intake (gm/day) of SD rats exposed to methanol extract of *R. mucronata* leaves. Low dose, 100 mg/kg, mid dose, 200 mg/kg, and high dose, 300 mg/kg.

The low dose 100 mg/kg administrated rats' water intake percentage ranges 97.2 ± 6.40 on 1^st^ day, 97.4 ± 8.50 on 7^th^ day, 97.6 ± 1.14 on 14^th^ day, 98.2 ± 1.40 on 21^st^ day, and 98.4 ± 1.50 on 28^th^ day. The mid dose 200 mg/kg administrated rats' water intake ranges 95.4 ± 2.10 on 1^st^ day, 95.3 ± 2.24 on 7^th^ day, 95.12 ± 2.02 on 14^th^ day, 96.4 ± 2.17 on 21^st^ day, and 96.5 ± 1.32 on 28^th^ day. The high dose 300 mg/kg administrated rats' water intake ranges 87.1 ± 1.30 on 1^st^ day, 87.2 ± 2.20 on 7^th^ day, 87.7 ± 4.52 on 14^th^ day, 87.2 ± 1.82 on 21^st^ day, and 87.4 ± 6.78 on 28^th^ day (Figure 5). The rise in water consumption from day 1 to day 28 suggests that all of the test animals drank enough water to satisfy their demands.

**Figure 6:**
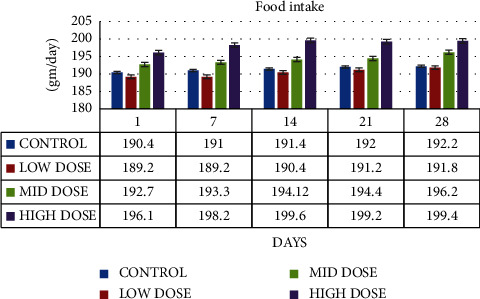
Graph shows the food intake (gm/day) of SD rats exposed to methanol extract of *R. mucronata* leaves.

The food intake of low dose 100 mg/kg administrated rats' ranges 189.2 ± 1.12 on 1^st^ day, 189.2 ± 1.02 on 7^th^ day, 190.4 ± 4.14 on 14^th^ day, 191.2 ± 2.12 on 21^st^ day, and 191.8 ± 1.22 on 28^th^ day. The mid dose 200 mg/kg administrated rats' food intake ranges 192.7 ± 4.33 on 1^st^ day, 193.3 ± 3.21 on 7^th^ day, 194.12 ± 2.22 on 14^th^ day, 194.4 ± 1.02 on 21^st^ day, and 196.2 ± 2.22 on 28^th^ day. The high dose 300 mg/kg rats' food intake ranges 196.1 ± 1.20 on 1^st^ day, 198.2 ± 2.21 on 7^th^ day, 199.6 ± 2.40 on 14^th^ day, 199.2 ± 1.20 on 21^st^ day, and 199.4 ± 2.45 on 28^th^ day (Figure 6).

**Figure 7:**
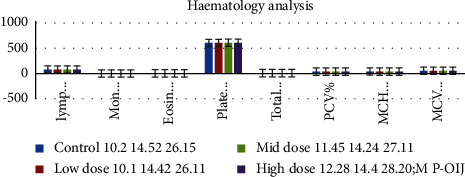
Graph shows the haematology observation of SD rats exposed to methanol extract of *R. mucronata* leaves. Low dose, 100 mg/kg, mid dose, 200 mg/kg, and high dose, 300 mg/kg.

Haematology results show the platelets counted were 604.16 ± 2.6610^3^/*µ*l in the control and 604.10 ± 4.26 10^3^/*µ*l in the low dose-, 607.42 ± 3.02 10^3^/*µ*l in the mid dose-, and 608.06 ± 4.54 10^3^/*µ*l in the high dose-treated animals (Figure 7). The haemoglobin content for the 100, 200, and 300 mg/kg treated animals was 10.10 ± 0.36 (g/dl), 11.45 ± 0.46 (g/dl), and 12.28 ± 0.26 (g/dl). The PVC, RBC, and WBC contents did not show any notable alterations.

**Figure 8:**
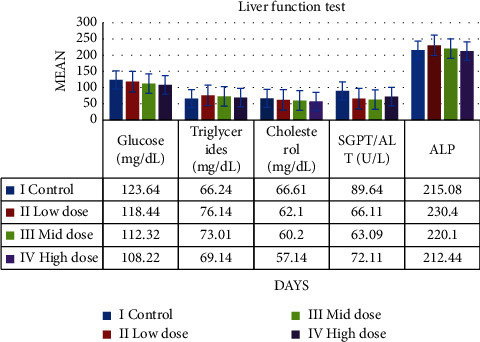
Graph shows the liver function parameters of SD rats exposed to methanol extract of *R. mucronata* leaves. Group I, control, group II, low dose (100 mg/kg), group III, mid dose (200 mg/kg), and group IV, low dose (300 mg/kg).

These liver function indicators, including total proteins, albumin, creatinine, triglycerides, cholesterol, alkaline phosphatase, and alanine amino transferase, were assessed. The level of liver function parameters decreases as extract dosage is raised. Rats treated with low, mid, and high doses as well as the control group showed no discernible differences.  Figure 8 shows the outcomes.

**Figure 9:**
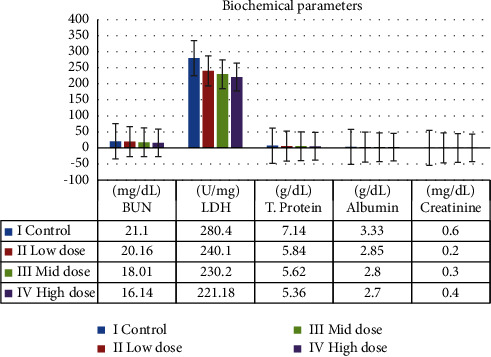
Graph shows the biochemical parameters of SD rats exposed to methanol extract of *R. mucronata* leaves. Group I, control, group II, low dose (100 mg/kg), group III, mid dose (200 mg/kg), and group IV, low dose (300 mg/kg).

The serum levels of the enzyme ALT, creatinine, and total protein did not vary significantly, according to the biochemical assays. The ALP value in MERML extract at 100 mg/kg demonstrated statistical significance up to 200 mg/kg. Despite this, Figure 9 shows the outcomes.

**Figure 10:**
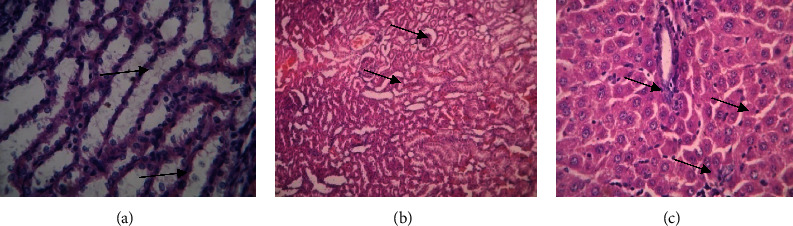
Histopathology images of low dose 100 mg/kg methanol extract-treated Sprague–Dawley rats group. (a) Kidney. (b) Liver. (c) Spleen. (a) Kidney-papillary ducts and glomerular capsule, (b) liver-hepatic cells and central veins. (c) Spleen, venus sinus, neutrophils, and lymphocytes.

**Figure 11:**
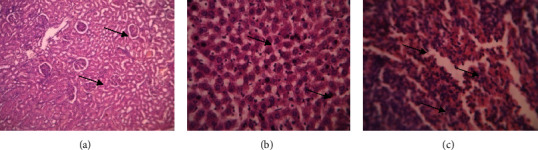
Histopathology images of mid dose 200 mg/kg methanol extract-treated Sprague–Dawley rats group. (a) Kidney. (b) Liver. (c) Spleen. (a) Kidney-papillary ducts and glomerular capsule, (b) liver-hepatic cells and central veins. (c) Spleen, venus sinus, neutrophils, and lymphocytes.

**Figure 12:**
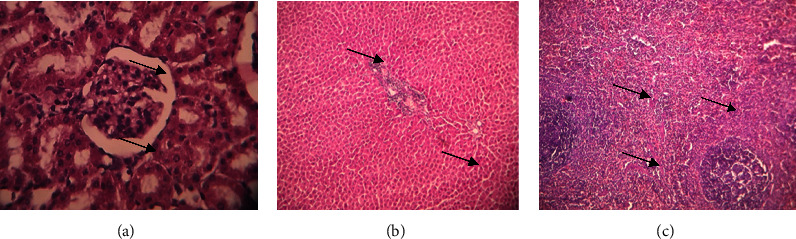
Histopathology images of high dose 300 mg/kg methanol extract-treated Sprague–Dawley rats group. (a) Kidney. (b) Liver. (c) Spleen. (a) Kidney-papillary ducts and glomerular capsule, (b) liver-hepatic cells and central veins. (c) Spleen, venus sinus, neutrophils, and lymphocytes.

The kidney, liver, and spleen histology examinations revealed no aberrant tissue, and many neutrophils and lymphocytes were also seen. This study states that there was no toxicity to any of the animals, for the whole 28-day period. Organ portions are shown in photographs in Figures 10–12.

### 3.3. *In Vivo* Anticancer Studies

*In vivo* anticancer effectiveness of MERML was studied using Sprague–Dawley rats. Group I animals serve as the control, DMBA (25 mg/kg) was used to induce breast cancer for the animals in Group II, 250 mg/kg of MERML extract was used in Group III, and 20 mg/kg of standard fluorouracil was used in Group IV.

#### 3.3.1. Final Body Weight

**Figure 13:**
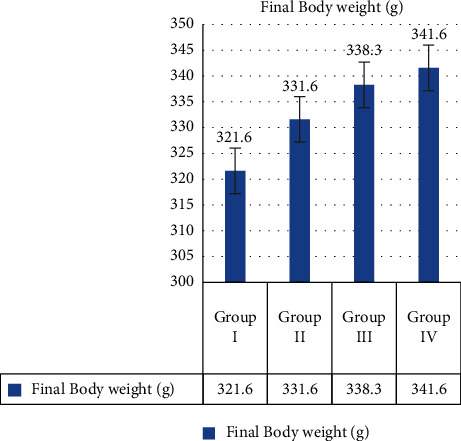
Graph shows the effect of body weight of SD rats exposed to methanol extract of *R. mucronata* leaves. Group I, control, group II, DMBA induced (25 mg/kg), group III, extract treated (250 mg/kg), and group IV, standard fluorouracil (200 mg/kg).

After receiving MERML extract, the weight of the test animals was noted. The control group rats were found to be 321.6 ± 2.88 gm, the DMBA cancer caused rats were found to be 331.6 ± 2.88 gm, the 250 mg/kg MERML extract treated animals were found to be 338.3 ± 2.88 gm, and the standard fluorouracil treated rats were found to be 341.6 ± 2.88 gm. The results are depicted in Figure 13. When compared to the Group II DMBA-induced animals, the body weight of the Group III animals has increased.

#### 3.3.2. Haematological Assays

**Figure 14:**
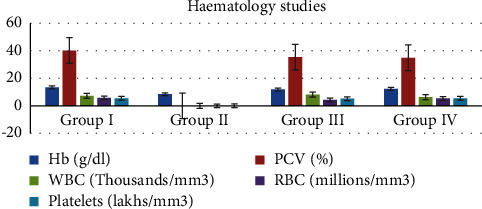
Graph shows the effect of haematology studies of SD rats exposed to methanol extract of *R. mucronata* leaves. Group I, control, group II, DMBA induced (25 mg/kg), group III, extract treated (250 mg/kg), and group IV, standard fluorouracil (200 mg/kg).

For both the control group and the groups that received treatment with the MERML extract, the haematological parameters including haemoglobin (Hb), red blood cells (RBCs), white blood cells (WBCs), packed cell volume (PCV), and platelet count were determined. The results are recorded and shown in Figure 14. 250 mg/kg methanolic extract treated Group III shows 11.83 ± 0.25 Hb level, 35.4 ± 0.90 PCV level, 8.16 ± 0.35 WBC level, 4.26 ± 0.20 RBC level, and 5.13 ± 0.35 platelets level. The treated Group III has a noticeable rise in the haematology analysis as compared to the Group II DMBA-induced.

#### 3.3.3. Serum Liver Function Markers

**Figure 15:**
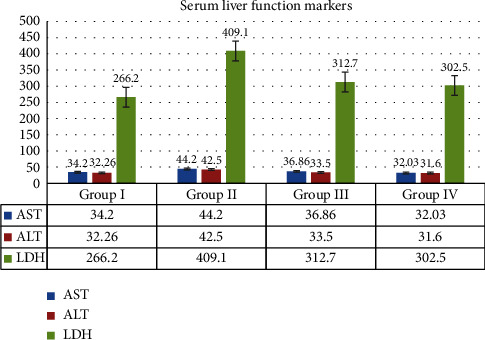
Graph shows the effect of serum liver function markers of SD rats exposed to methanol extract of *R. mucronata* leaves. Group I, control, group II, DMBA induced (25 mg/kg), group III, extract treated (250 mg/kg), and group IV, standard fluorouracil (200 mg/kg).

For all the animals in Groups I, II, III, and IV, the biochemical parameters of serum analysis for the liver function markers were examined. The 250 mg/kg MERML extract treated group depicts the aspartate transaminase activity at 36.86 ± 2.91, alanine transaminase activity at 33.5 ± 2.16, and lactate dehydrogenase activity at 312.7 ± 8.87. The assays are estimated and shown in Figure 15. When compared to the Group II DMBA induced, the treated Group III has significant decrease in the AST, ALT, and LDH activity.

#### 3.3.4. Serum Renal Function Markers

**Figure 16:**
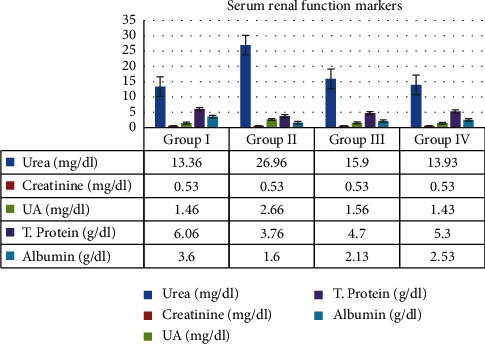
Graph shows the effect of serum renal function markers of SD rats exposed to methanol extract of *R. mucronata* leaves. Group I, control, group II, DMBA induced (25 mg/kg), group III, extract treated (250 mg/kg), and group IV, standard fluorouracil (200 mg/kg).

All the animals in Groups I, II, III, and IV had their biochemical kidney function markers examined. When compared to the control, 13.36 ± 0.15, 1.46 ± 0.15, and standard control groups 13.93 ± 0.63, 1.43 ± 0.05, the treated groups have higher values of urea (15.9 ± 0.72, 1.56 ± 0.20), and uric acid (13.36 ± 0.15, 1.46 ± 0.15). But, there is a decrease in total proteins 4.7 ± 0.17 and albumin 2.13 ± 0.11 when compared to the control, 6.06 ± 0.23, 3.60 ± 0.34, and standard groups, 5.30 ± 0.17, 2.53 ± 0.25. Creatinine level, 0.53 ± 0.11, does not show any significant changes from the control, 0.53 ± 0.05 and standard control groups, 0.53 ± 0.05. The results are estimated and shown in Figure 16. When compared to the DMBA induced Group II, the treated Group III has significant increase in total protein and albumin and decrease in urea and uric acid. Creatinine level does not show the major changes in both Group II and Group III animals.

#### 3.3.5. Serum Enzymatic and Nonenzymatic Antioxidant Enzymes

All of the animals in Groups I, II, III, and IV had their serum enzymatic and nonenzymatic antioxidant assays performed to determine their biochemical characteristics. The superoxide dismutase is a key enzyme in the oxidative stress which has the capacity of scavenging further range of liberated radicals. SOD observed in the treated group was 5.16 ± 0.35, and in the standard control group, it was 6.36 ± 0.15; in the control group, it was 7.43 ± 0.20, and in the DMBA induced group, it was 3.60 ± 0.10. Thus, the treated Group III exhibits a significantly higher level of superoxide dismutase activity when compared to DMBA-induced Group II.

Catalase is an antioxidant enzyme, which protects the cell by splitting the two hydrogen peroxide molecules into single particle of oxygen. The MERML extract-treated animals show 34.13 ± 1.70 and the standard control group shows 36.60 ± 1.11 values. In the control and DMBA-induced groups, the catalyse enzyme activity shows 37.03 ± 1.33 and 26.93 ± 1.30 values. The MERML-treated Group III has a considerable increase in catalase activity when compared to the DMBA-induced Group II.

Glutathione peroxidase activity shows 5.83 ± 0.35 in the treated groups and 6.90 ± 0.26 in the standard control groups correspondingly. In the control groups, GPX activity shows 8.50 ± 0.30, and in the DMBA induced groups, GPX activity shows 3.43 ± 0.15. The GPX activity of MERML treated groups is increased when compared to the DMBA induced groups. There is a small dissimilarity observed among the control groups when compared with treated groups.

**Figure 17:**
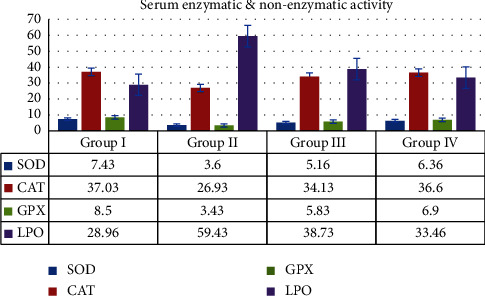
Graph shows the effect of serum enzymatic and nonenzymatic antioxidant activities of SD rats exposed to methanol extract of *R. mucronata* leaves. Group I, control, group II, DMBA induced (25 mg/kg), group III, extract treated (250 mg/kg), and group IV, standard fluorouracil (200 mg/kg).

Lipid per oxidation assay is used to assess the oxidative stress. In the MERML extract treated groups, the LPO values show 38.73 ± 4.35, and in standard control treated group, it shows 33.46 ± 1.36. In the control groups, the value is 28.96 ± 3.30, and in the DMBA induced groups, it is 59.43 ± 2.72. When compared to the DMBA-induced groups, the MERML treated groups' LPO activity is lower.  Figure 17 displays the estimated antioxidant levels.

#### 3.3.6. Histopathological Investigations

**Figure 18:**
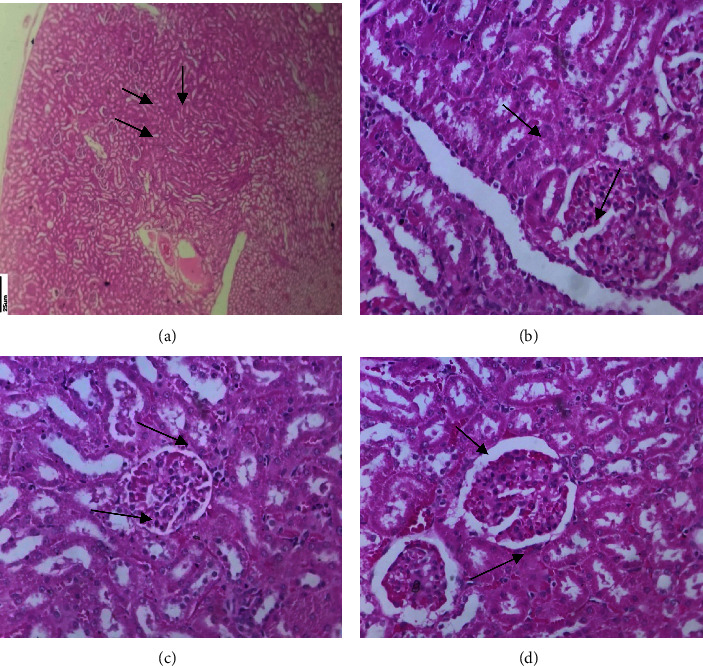
Histopathology analysis of kidney: (a) control, (b) 20 mg/kg DMBA induced, (c) methanolic extract of *R. mucronata* 250 mg/kg treated, and (d) 20 mg/kg fluorouracil standard control. (a) Normal cortex, medulla. (b) Intact tubules surrounded, glomeruli, and interstitial inflammation with necorsis. (c) Normal cells and mild interstitial inflammation. (d) Normal cells and mild inflammation.

In control groups, the histological section of the kidney shows a normal cortex with a normal medulla region. There is evidence of necrosis and glomeruli surrounded by intact tubules in the 25 mg/kg DMBA cancer-induced group. Tissue sections from the 250 mg/kg MERML-treated groups show normal and mild inflammation. Also, there is evidence for normal and mild inflammation in the 20 mg/kg fluorouracil standard control group. The findings demonstrate that the DMBA cancer inducer promotes necrosis in Group II rats but not in MERML extract-treated Group III rats or standard control Group IV rats.  Figure 18 depicts the study of kidney sections.

**Figure 19:**
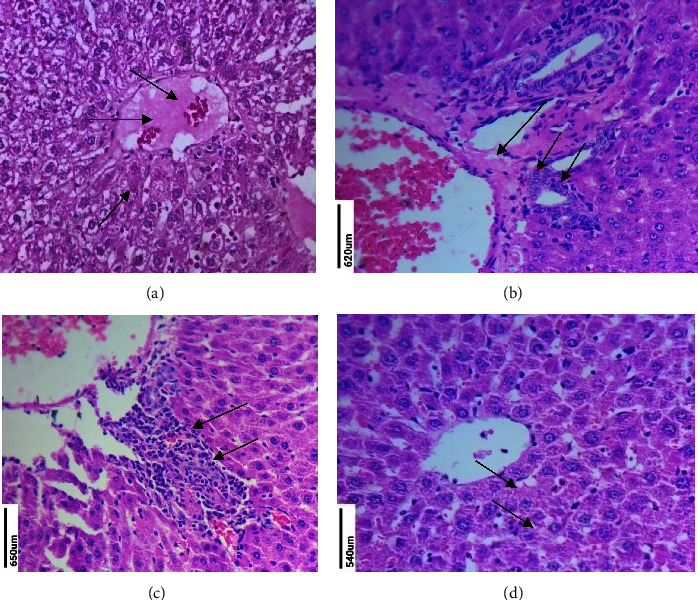
Histopathology analysis of liver: (a) control, (b) 20 mg/kg DMBA induced, (c) methanolic extract of *R. mucronata* 250 mg/kg treated, and (d) 20 mg/kg fluorouracil standard control. (a) Normal hepatocytes with normal central vein. (b) Abnormal lobules, inflammation, necrosis, congestion, and loss of some liver parenchymal cells. (c) Normal central vein with mild inflammation. (d) Normal central vein and normal sinusoids.

In the control groups, tissue biopsies of the liver show normal hepatocytes with a normal central vein. The aberrant lobules with focal area of fatty alteration, inflammation, necrosis, congestion, and loss of certain liver parenchymal cells can be seen in the 25 mg/kg DMBA cancer caused group. The 250 mg/kg MERML extract-treated Group III showed a normal central vein with mild inflammation. The 20 mg/kg fluorouracil standard control shows the normal central vein and sinusoids are normal. The findings reveal that DMBA cancer inducer promotes inflammation, necrosis, and cell death in Group II rats, but not in MERML extract-treated Group III or standard control Group IV rats.  Figure 19 depicts the study of liver sections.

**Figure 20:**
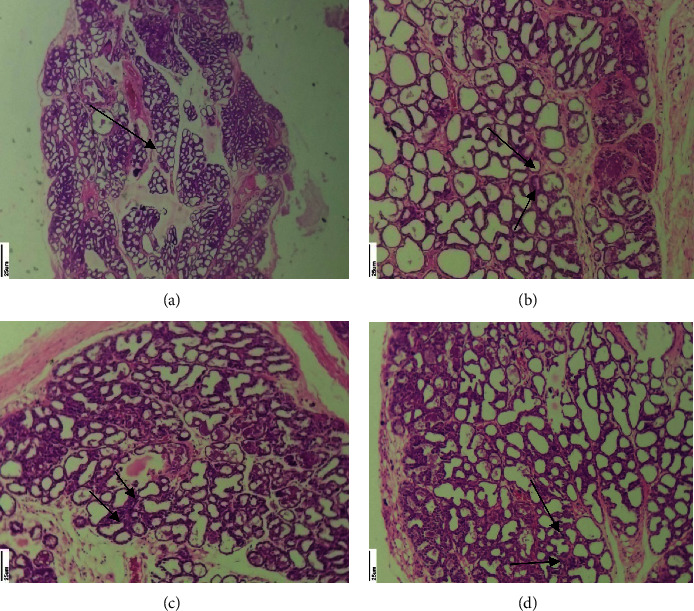
Histopathology analysis of mammary tissue: (a) control, (b) 20 mg/kg DMBA induced, (c) methanolic extract of *R. mucronata* 250 mg/kg treated, and (d) 20 mg/kg fluorouracil standard control. (a) Normal duct lobule units with fatty tissues. (b) High mitotic activity with the necrosis and reactive lymphatic follicular hyperplasia. (c) Hyperchromasia. (d) Terminal duct lobule with some neoplastic cells.

The normal duct lobule units with fatty tissues are visible in the mammary tissue of Group I control rats. High mitotic activity, necrosis, and reactive lymphatic follicular hyperplasia were found in the 25 mg/kg DMBA cancer-induced Group II rats. In the mammary tissue of rats treated with 250 mg/kg MERML extract, the typical arrangement of the cells in glandular pattern, which implies nuclear hyperchromasia, was discovered. The tissue section of 20 mg/kg fluorouracil standard control rats revealed terminal duct lobule units as well as some neoplastic cells. The findings reveal that the DMBA cancer inducer promotes necrosis and benign tumours in Group II rats, but the MERML inhibits cancer cell growth while displaying darker nuclei in the Group III rats.  Figure 20 depicts the results of mammary tissue sections.

## 4. Discussion

In the current investigation, an acute toxicity test was conducted in animals to determine if the administration of MERML had any toxicity effects with behavioural changes, body weight, and food and drink intake. At the end of the experimental study over the following 14 days, there were no appreciable differences between the treatment groups and the control group in the general behaviour, weight, or food intake of the rats. It is hypothesised that rats' normal growth was influenced by the acute oral doses of MERML. The authors of [8, 9] state that changes in body weight have been indicted as a sign of adverse pharmacological and chemical effects.

According to [10], measuring food and water intake is important when examining the safety of a product intended for therapeutic purposes because an animal's physiological status and the accomplishment of the proper reaction to the medications being tested depend on an adequate intake of nutrients. Over the extent of our study, there was no discernible change in body weight, although usual amounts of food and water were consumed.

The authors of [11, 12] examined the toxicity profile of shade-dried *Orthosiphon stamineus* leaves and discovered that the plant did not exhibit toxicological symptoms in rodents. The results of the current investigation proved that methanolic extract of *Rhizophora mucronata* leaves can be used safely. Both the control and the MERML test groups had normal values for body weight, posture, body tone, piloerection, defecation, sensitivity response, locomotion, muscular grip strength, and urination. Convulsion-related limb paralysis symptoms are not present in the MERML experimental groups and are normal in the control groups. There was no evidence of toxicity or rat death in the oral acute toxicity trials of MERML up to the dose of 1000 mg/kg body weight. According to [13], drugs with an oral LD_50_ greater than 5000 mg/kg may be regarded as nontoxic. As a result, MERML used orally are nontoxic.

In [14], it is said that there were no significant changes in the weight of the body and organs after a 28-day therapy with methanolic leaves extract of *T. citrina.* In our study, three dose levels, low 100 mg/kg, mid 200 mg/kg, and high 300 mg/kg of MERML, were administered to animals during the subacute oral toxicity assessment. Each animal showed a typical increase in body weight with no significant differences between the control and treatment groups. Animals in the low dose, mid dose, and high dose treatment groups in our study did not exhibit any appreciable alterations.

According to [15], the haematopoietic system is one of the important indicators of clinical and physiological status in rats. The change in the test-treated and control animals was visible in the RBC records, MCV, MCH, and MCHC. The importance of determined blood indices in analysing weaknesses has been taken into consideration [16]. In our study, the haematological parameters between the control and MERML treated groups demonstrated that the MERML had no changes in the values of platelets or red blood cells.

The histopathological studies of *Terminalia chebula* on the spleen, liver, and kidney have lately demonstrated similar actions and have been used as excellent therapeutic agents, as suggested by [17]. In our study, the histological analysis of the organs further supported these impressions. Based on the findings, we came to the conclusion that the MERML was nontoxic, safer, and suitable for pharmacological and therapeutic uses.

According to [18], Wistar rats that were 50–56 days old were administered a single dosage of 80 mg/kg DMBA intragastrically to stimulate mammary growths. Rats in this age range become extremely vulnerable to cancer-causing substances and tumour growth because of the active expansion of the terminal ducts in breast tissue. This result is consistent with studies [19] that found similar results with DMBA in female Sprague–Dawley rats. In our work, breast cancer in SD rats was induced by DMBA.

In our study, the ability of the MERML to reduce the high levels of ALP in DMBA-induced rats suggests that it may be able to stabilise and exchange metabolites across cell membranes and the fusion of protein and glycogen metabolism. Also, the treatment with MERML inhibits DMBA-induced rats, and the difference appears to be statistically significant (*p* < 0.05). The high levels of ALP found in cancer-induced animals are feature to tumour and metastasis. The actions of liver marker enzymes are related to the level of tumour growth and can be used as a sign for analysis of cancer. The activity of liver marker enzymes is significantly restored in MERML treated mice when compared with tumour induced Group II animals to that of normal control Group I animals. Furthermore, *A. comosus* peel extract is also proved to ameliorate the effect of DMBA induced mammary carcinoma [21].

Zingue et al. [22] demonstrated that DMBA alters the normal process of mammary gland differentiation of terminal ducts into alveoli and lobules. In our study, the DMBA-induced mammary tissue sections show the differentiation of terminal ducts. Additionally, the histological analysis of MERML treatment animals' mammary tissue sections shows the adverse effect in inhibiting the growth of cancer cells. However, the kidney and liver tissue sections did not show any significant changes.

The *in vivo* anticancerous properties of the methanolic leaves extract of *Rhizophora mucronata* (MERML) are well analysed. Notably, flavonols present in the MERML exhibits the cancer-preventive properties for targeting mammary gland tissue. The bioactive component present in the methanolic leaf extract of *Rhizophora mucronata* may be a viable candidate for the development of a pharmaceutically effective breast cancer treatment medication.

## 5. Conclusion

In conclusion, throughout the experimental procedures, there was no mortality of Sprague–Dawley rats, and no significant alterations were seen in the control and MERML dose-treated groups in the acute, subacute, and anticancer investigation. This established that MERML does not show the toxicity to the animals and it is safe to use. Due to the presence of various phytochemicals, MERML extract shows a competent antagonistic effect against breast cancer. Based on the *in vivo* testing, it was recognized that the methanolic leaves extract of *Rhizophora mucronata* has the influence to inhibit the growth of breast cancer. Furthermore, the isolation and purification of bioactive substances from *Rhizophora mucronata* leaves may reveal the presence of potent anticancer compound. Thus, *Rhizophora mucronata* leaves possess anticancer properties against the breast cancer in the field of herbal medicine.

## Figures and Tables

**Table 1:** The animal groups specification in acute studies.

S. no.	Groups	Specifications	No. of rats
1	Group I	Control rats received normal pelleted diet	6 females
2	Group II	*R. mucronata*-high dose X (1000 mg)	6 females

**Table 2:** The animal groups specification in subacute studies.

S. no.	Groups	Specifications	No. of rats
1	Group I	Control rats received normal pelleted diet	6 females
2	Group II	*R. mucronata*-low dose X (100 mg)	6 females
3	Group III	*R. mucronata*-mid dose 2X (200 mg)	6 females
4	Group IV	*R. mucronata*-high dose 3X (300 mg)	6 females

**Table 3:** The animal groups specification in anticancer studies.

S. no.	Groups	Specifications	No. of rats
1	Group I	Control rats received normal pelleted diet	6 females
2	Group II	Mammary carcinoma was induced in animals by a single dose of DMBA 20 mg/kg (7,12-dimethylbenz(a)anthracene)	6 females
3	Group III	Test sample, mammary carcinoma rats treated with methanolic extract of *R. mucronata* 250 mg/kg body weight	6 females
4	Group IV	Positive control, mammary carcinoma rats treated with 20 mg/kg standard drug (5′fluorouracil, Sigma-Aldrich)	6 females

**Table 4:** The behavioural indications of group I and group II animals.

No.	Dose (mg/kg)	1	2	3	4	5	6	7	8	9	10	11	12	13	14	15	16	17	18	19	20
1	Control	+	−	−	+	−	+	−	−	−	−	−	−	−	−	−	−	−	−	+	−
2	1000	+	−	−	+	−	+	−	−	−	−	−	−	−	−	−	−	−	−	+	−

1. alertness; 2. aggressiveness; 3. pile erection; 4. grooming; 5. gripping; 6. touch response; 7. decreased motor activity; 8. tremors; 9. convulsions; 10. muscle spasm; 11. catatonia; 12. muscle-relaxant; 13. hypnosis; 14. analgesia; 15. lacrimation; 16. exophthalmos; 17. diarrhoea; 18. writhing; 19. respiration; 20. mortality.

## Data Availability

The datasets generated during and/or analysed during the current study are available from the corresponding author on reasonable request.
